# Is the anatomical distribution of low-grade gliomas linked to regions of gliogenesis?

**DOI:** 10.1007/s11060-020-03409-8

**Published:** 2020-01-25

**Authors:** Anne Jarstein Skjulsvik, Hans Kristian Bø, Asgeir Store Jakola, Erik Magnus Berntsen, Lars Eirik Bø, Ingerid Reinertsen, Kristin Smistad Myrmel, Kristin Sjåvik, Kristin Åberg, Thomas Berg, Hong Yan Dai, Roar Kloster, Sverre Helge Torp, Ole Solheim

**Affiliations:** 1 Department of Pathology, St. Olavs University Hospital, Trondheim, Norway; 2 Departments of Clinical and Molecular Medicine, Faculty of Medicine, NTNU, Norwegian University of Science and Technology, 7491 Trondheim, Norway; 3 Department of Diagnostic Imaging, Nordland Hospital Trust, Bodø, Norway; 4 Department of Circulation and Medical Imaging, Faculty of Medicine, NTNU, Norwegian University of Science and Technology, 7491 Trondheim, Norway; 5 Department of Neurosurgery, Sahlgrenska University Hospital, Gothenburg, Sweden; 6 Institute of Neuroscience and Physiology, Sahlgrenska Academy, Gothenburg, Sweden; 7 Department of Neurosurgery, St. Olavs University Hospital, Olav Kyrres Gate, 7006 Trondheim, Norway; 8 Department of Radiology and Nuclear Medicine, St. Olavs University Hospital, Olav Kyrres Gate, 7006 Trondheim, Norway; 9 Department of Health Research, SINTEF Digital, Trondheim, Norway; 10 Department of Clinical Pathology, University Hospital of North Norway, Tromsö, Norway; 11 Department of Neurosurgery, University Hospital of North Norway, Tromsö, Norway; 12 Department of Neuroscience and Movement Medicine, Faculty of Medicine, NTNU, Norwegian University of Science and Technology, 7491 Trondheim, Norway

**Keywords:** Low-grade glioma, Subventricular zone, Subgranular zone, IDH mutation, 1p19q co-deletion, 3D brain map

## Abstract

**Introduction:**

According to the stem cell theory, two neurogenic niches in the adult human brain may harbor cells that initiate the formation of gliomas: The larger subventricular zone (SVZ) and the subgranular zone (SGZ) in the hippocampus. We wanted to explore whether defining molecular markers in low-grade gliomas (LGG; WHO grade II) are related to distance to the neurogenic niches.

**Methods:**

Patients treated at two Norwegian university hospitals with population-based referral were included. Eligible patients had histopathological verified supratentorial low-grade glioma. IDH mutational status and 1p19q co-deletion status was retrospectively assessed. 159 patients were included, and semi-automatic tumor segmentation was done from pre-treatment T2-weighted (T2W) or Fluid-Attenuated Inversion Recovery (FLAIR) images. 3D maps showing the anatomical distribution of the tumors were then created for each of the three molecular subtypes (IDH mutated/1p19q co-deleted, IDH mutated and IDH wild-type). Both distance from tumor center and tumor border to the neurogenic niches were recorded.

**Results:**

In this population-based cohort of previously untreated low-grade gliomas, we found that low-grade gliomas are more often found closer to the SVZ than the SGZ, but IDH wild-type tumors are more often found near SGZ.

**Conclusion:**

Our study suggests that the stem cell origin of IDH wild-type and IDH mutated low-grade gliomas may be different.

## Introduction

The origin of gliomas, and the question of whether all gliomas have a similar or even the same cellular origin, is much discussed. The long-reigning hypothesis that gliomas arise from fully differentiated glia has been largely abandoned and it is now widely believed that cancer stem cells contribute to gliomagenesis and treatment resistance [1–4].

Two niches in the adult human brain harbor neural stem cells (NSC) that ensures active neurogenesis and glial cell migration: the larger subventricular zone (SVZ) and the subgranular zone (SGZ) in the hippocampus [5]. Due to their shared genetic expression with tumor cells, capability of self-renewal and migratory potential the NSCs might be capable of transformation into cancer stem cells and initiate glioma formation [6–8]. Identifying a cell of origin is fundamental for understanding disease progression, and a recent study by Lee et al. speculated that astrocyte-like NSCs with low-level driver mutations (TERT, EGFR, PTEN and TP53) in the SVZ may be the cell of origin in glioblastomas [8].

A defining event in the development and progression of gliomas is the isocitrate dehydrogenase (IDH) mutation [9], which is present in 70% of low-grade gliomas [10]. It precedes 1p19q co-deletion, which is also considered an early event in gliomagenesis [11, 12]. In the recent WHO classification these two molecular markers now define the diffuse glioma subtypes with different prognoses. Oncogenic mutations in stem cells or progenitor cells might also affect cancer cell migration and several studies have reported that the mutational status is related to the anatomical distribution pattern of low-grade glioma (LGG) subtypes in the brain. We therefore hypothesized that the anatomical distribution of LGG molecular subtypes may relate to the neurogenic niches.

In this population-based study with data from two Norwegian university hospitals, we aimed to analyze the anatomical distribution of LGGs of WHO grade II in a three-dimensional (3D) standard Montreal Neurological Institute (MNI) space. We wanted to explore whether molecular subtype could be linked to distance from lesions to the two neurogenic niches in human adults, namely the subventricular zone and the subgranular zone.

## Materials and methods

### Patients

Eligible patients were adults (≥ 18 years) diagnosed with supratentorial LGG at St. Olavs University Hospital between 1998 and 2015 or at the University Hospital of North Norway between 1998 and 2009. The two university hospitals have region-based referral and together cover a geographical catchment region with 1.2 million inhabitants (Statistics Norway; https://www.ssb.no/en/). Both hospitals favored early histopathological diagnosis (diagnostic biopsy or surgical resection) in suspected LGGs. Diagnostic MRIs were retrieved, and tissue samples were revised and analyzed to assess IDH status and 1p19q status. Of a total of 205 patients, 66 patients were diagnosed at the University Hospital of North Norway while the remaining 139 patients were diagnosed at St. Olavs University Hospital. 39 patients were excluded as diagnostic MRIs were unavailable or incomplete, six patients were excluded due to the lack of tissue to perform retrospective molecular analyses and one patient was excluded due to a change in diagnosis after histopathological review. Our final sample included 159 LGGs (62 from the University Hospital of North Norway and 97 patients from St. Olavs University Hospital).

### Radiology and image interpretation

A radiologist experienced with LGG assessment and segmentation (H.K.B.) performed semi-automatic tumor segmentation as previously described [13]. Segmentations were performed in the open source software 3D Slicer 4.4.0 (https://www.slicer.org), a software platform for quantitative imaging [14]. Images were acquired on 18 different MRI systems on 10 different locations with field strengths between 0.5 and 3.0 T (16 on 0.5 T, 4 on 1.0 T, 86 on 1.5 T and 53 on 3.0 T). Of the MRI examinations used for pre-operative tumor segmentation 122 were Fluid-Attenuated Inversion Recovery (FLAIR) images, 30 were T2-weighted images and seven were T1-weighted contrast enhanced images. 92 of the 159 acquisitions were 3D sequences with a slice thickness of 1.0–2.0 mm and no inter-slice gap. 67 of 159 acquisitions were two- dimensional (2D) sequences with slice-thickness of 2.5–6.5 mm and inter-slice gap of 0.25–2.0 mm. In-plane resolution was in the range 0.41–1.02 mm (mean 0.88 mm).

### Image registration

The segmented MRI images were all registered to the standard MNI coordinate space, which is defined by the ICBM-152 brain template [15]. The registration pipeline, which was based on the Advanced Normalization Tools (ANTs) toolkit [16], has been described in detail in a previous paper [17]. The registrations were controlled manually, and in five cases they were corrected, either by modifying registration parameters (three cases) or by applying a manual, landmark-based registration (two cases). The registered segmentations were then summed voxel-wise to create maps of the tumor distribution.

Surfaces representing the SVZ were created manually based on the description given by Vescovi et al. [2] and the rendering of the lateral ventricles given in the Hammersmith atlas [18]. This atlas complies with the MNI space, and the distances from the registered tumors to the SVZ could thus be calculated directly.

The location of the SGZ was defined using the high-resolution atlas of the hippocampus and subfields created by the CoBrA Lab [19], which is available at https://cobralab.ca/atlases/Hippocampus-subfields/. The atlas was first registered to the MNI space, and the location of the SGZ was then defined as the center of mass of the dentate gyrus.

### Molecular markers

The IDH mutational status and 1p19q co-deletion status were analyzed as previously described [20]. In summary, based on molecular markers patients were classified into three molecular groups: (i) the low-risk group being IDH mutated, 1p19q co-deleted, (ii) the intermediate-risk group being IDH mutated and 1p19q non-co-deleted, and (iii) the high-risk group being IDH wild-type [21]. These risk groups correspond to the WHO classification of (i) oligodendroglioma, IDH-mutant and 1p19q co-deleted, (ii) diffuse astrocytoma, IDH-mutant and (iii) diffuse astrocytoma, IDH wild-type.

Different diagnostic approaches were used at the two hospitals. For 62 patients the 1p19q co-deletion and IDH status were determined using multiplex ligation-dependent probe amplification (MLPA) directly since there was limited amount of tissue available. Samples classified as IDH wild-type after MLPA assessment were subject to PCR and DNA sequencing for IDH1 and IDH2 mutations. For 97 patients an integrated approach was used. Here, immunohistochemistry for IDH1 R132H and alpha thalassemia/mental retardation syndrome X-linked (ATRX) protein expression was done as an initial step. If IDH R132H mutation and accompanying ATRX loss were observed, no further analyses were carried out and tumors were classified as IDH mutated, 1p19q non-co-deleted. Samples negative for IDHR132H mutation on immunohistochemistry were subject to PCR and DNA sequencing for other IDH1 and IDH2 mutations. In samples with IDH mutation and ATRX presence, we then carried out fluorescence in situ hybridization (FISH) to confirm the 1p19q codeletion. However, in three cases there was too little additional tissue available, and we had to assume 1p19q co-deletion based only IDH and ATRX presence without FISH confirmation. Also, for a selected group of IDH1 R132H-negative patients (n = 7) we were not able to do PCR and DNA sequencing for other IDH-mutations.

### Statistics

Analyses were performed using IBM SPSS Statistics for Windows, Version 25.0. Statistical significance was set to p < 0.05. Q-Q plots were used to assess normal distribution in continuous data. Chi-squared tests were used for comparison analysis of categorical variables. Nonparametric Mann–Whitney U and Kruskall-Wallis test were used for comparison of continuous variables.

## Ethics

The regional ethical committee of Central Norway approved this project (reference 2016/1377 and 2015/1460).

## Results

**Fig. 1:**
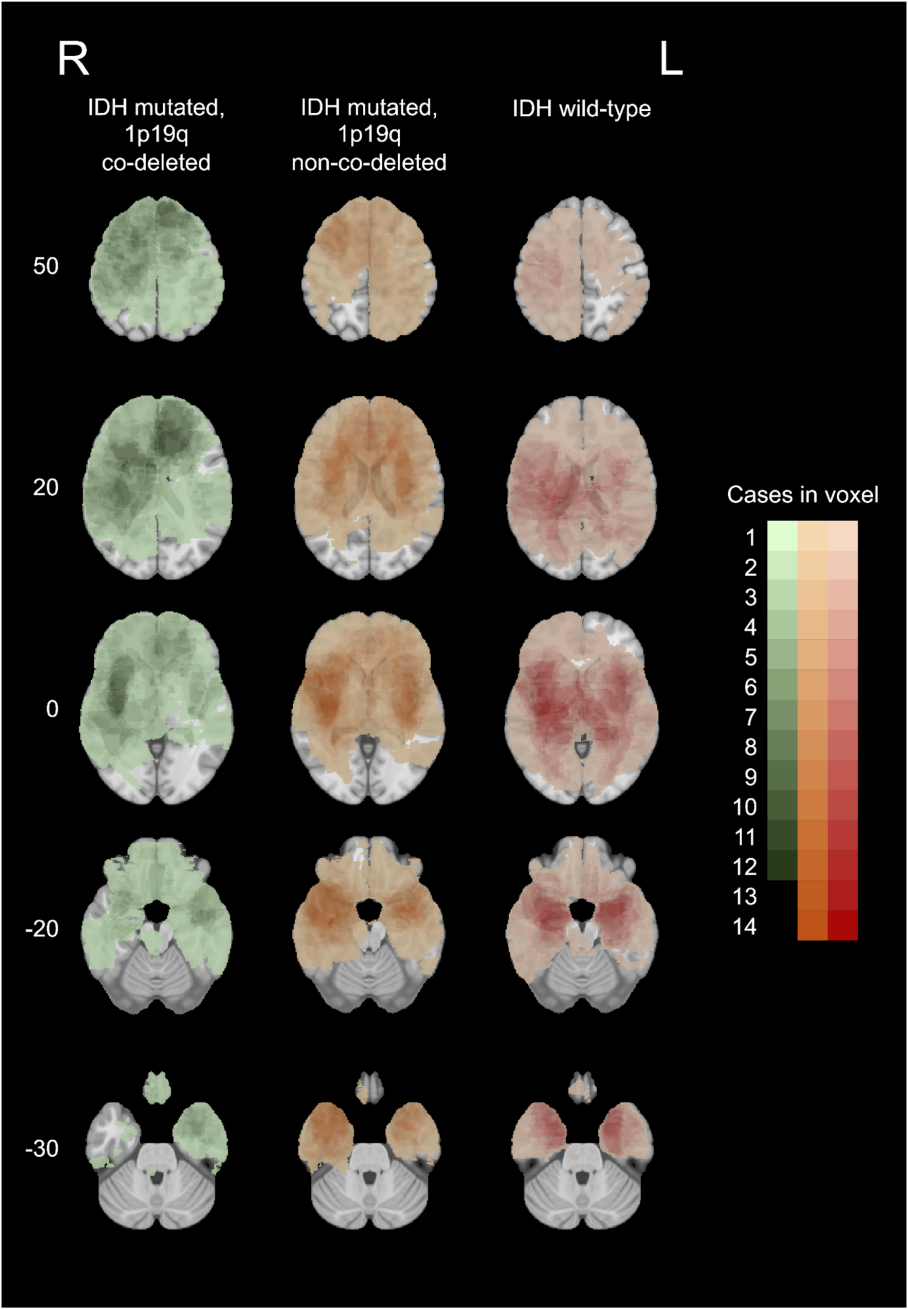
Heatmap of tumor showing distribution of the three molecular groups: oligodendrogliomas (IDH mutated, 1p19q co-deleted) in left panels (n = 50), astrocytomas (IDH mutated, 1p19q non-co-deleted) in middle panels (n = 64) and astrocytomas (IDH wild-type) in right panels (n = 45). Increasing intensity of color corresponds to increasing number of overlapping tumors within the same group. Numbers on left corresponds to z-coordinates in the MNI space and sections marked 0 and -20 represents upper and lower part of hippocampus, respectively

The study included 159 previously untreated patients with LGG. 113 were IDH mutated, 46 were IDH wild-type, and 49 were both IDH mutated and 1p19q co-deleted (Fig. 1; Table 1). Based on tumors segmented in pretreatment MRI registered to MNI space, we measured the shortest distance from tumor border and tumor center to both neurogenic niches (Table 2). In 108 patients, the tumor involved SVZ and in 72 patients the tumor involved SGZ and the distance from tumor border to neurogenic niches was set to zero.

**Table 1:** Patient and tumor characteristics

Patient characteristics	No of patients (%)
Mean age at surgery (SD)	46 (15)
Female gender (%)	58 (37)

Mutational status	No of patients (%)
IDH mutated	114 (72)
IDH wild-type	45 (28)
1p19q co-deleted	50 (31)

Molecular group	No (%)
1. Oligodendroglioma, IDH mutated, 1p19q co-deleted	50 (31)
2. Astrocytoma, IDH mutated, 1p19q non-co-deleted	64 (40)
3. Astrocytoma, IDH wild-type	45 (28)

Tumor volume	Median (IQR)
Oligodendroglioma, IDH mutated, 1p19q co-deleted	48.4 ml (78.9)
Astrocytoma, IDH mutated, 1p19q non-co-deleted	73.4 ml (92.8)
Astrocytoma, IDH wild-type	45.9 ml (115.3)

Relation to neurogenic niches	No (%)
Contact with SVZ	108 (68)
Contact with SGZ	72 (45)
Contact with both SVZ and SGZ	70 (44)
No contact with either SVZ or SGZ	48 (30)

**Table 2:** Median distance from neurogenic niches, both from border of tumor and center of tumor

	Subventricular zone		Subgranular zone	
	Median distance (Q1–Q3)		Median distance (Q1–Q3)	
	Border	Center	Border	Center
WHO Diagnosis/risk group				
1. Oligodendroglioma, IDH mutated, 1p19q co-deleted	0 mm (0–4.6)	20.7 mm (13.3–25.9)	15.4 mm (0–34.4)	42.3 mm (22.7–57.4)
2. Astrocytoma, IDH mutated, 1p19q non-co-deleted	0 mm (0–4.3)	16.5 mm (12.0–23.7)	3.8 mm (0–22.4)	31.4 mm (15.1–52.0)
3. Astrocytoma, IDH wild-type	0 mm (0–4.3)	14.8 mm (6.7–20.7)	0 mm (0–12.6)	20.8 mm (8.4–32.6)
IDH mutation status				
IDH mutated	0 mm (0–4.5)	18.7 mm (12.3–24.5)	7.7 mm (0–29.9)	35.7 mm (18.5–56.0)
IDH wild-type	0 mm (0–4.3)	14.9 mm (6.7–20.7)	0 mm (0–12.6)	20.8 mm (8.4–32.6)

**Fig. 2:**
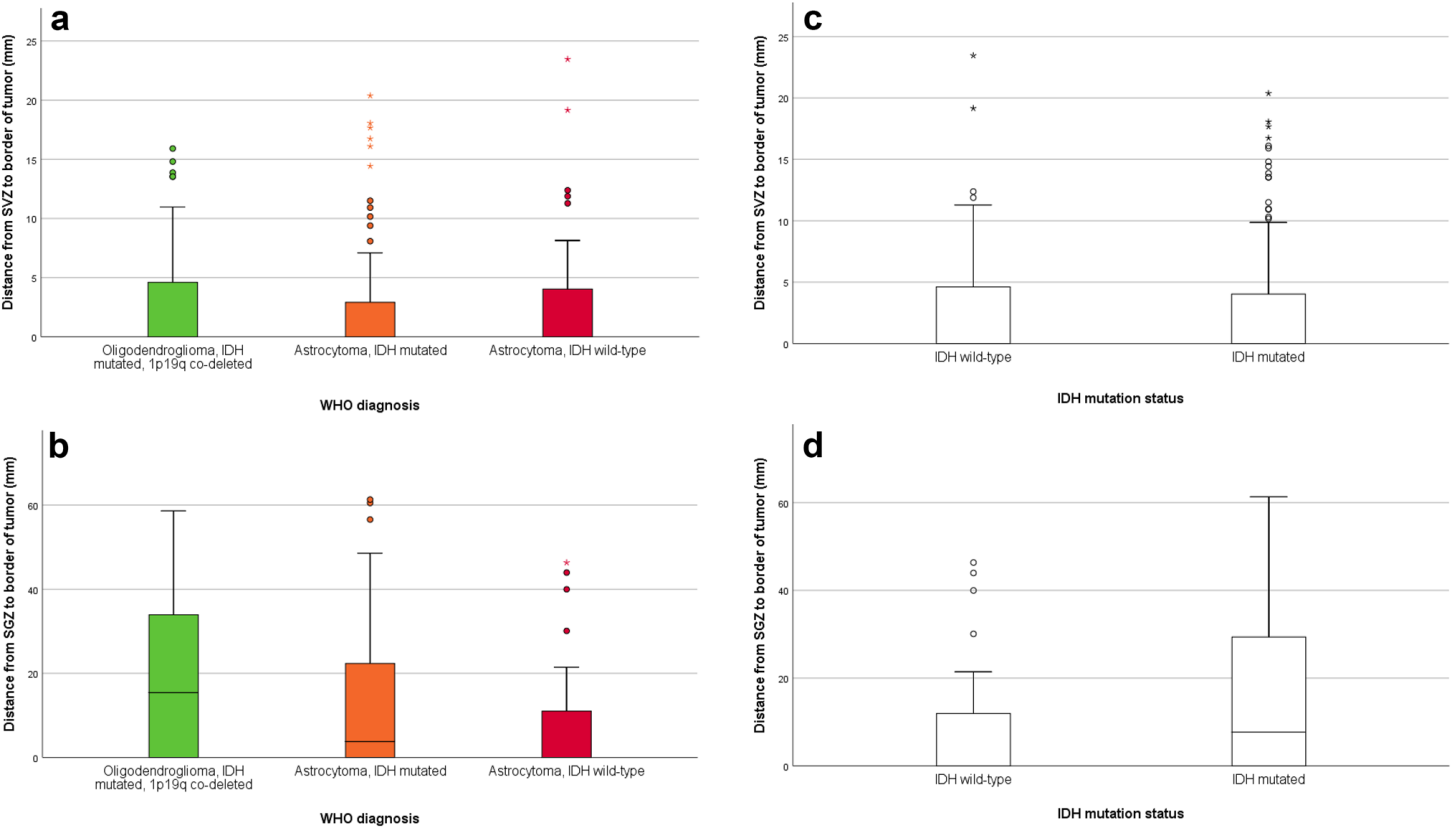
**a** Shortest distance in millimeters from the tumor border to the subventricular zone (SVZ) for the different groups of low-grade gliomas. **b** Shortest distance in millimeters from the tumor border to the subgranular zone (SGZ) for the different groups of low-grade gliomas. **c** Shortest distance in millimeters from the tumor border to the subventricular zone (SVZ) for different groups of IDH mutation status. **d** Shortest distance in millimeters from tumor border to the subgranular zone (SGZ) for different groups of IDH mutation status

There were differences between the different molecular groups (IDH mutated/1p19q co-deleted vs IDH mutated vs IDH wild-type) when it came to the distance from SVZ to tumor, but only when measured from tumor center (p = 0.033, median distance 20.7 mm vs 16.5 mm vs 14.8 mm) and not from tumor border (p = 0.797, median distance was 0 mm for all groups) (Fig. 2a). There were also differences between molecular groups when it came to distance to the SGZ, both from tumor center (p < 0.001, median distance 42.3 mm vs 31.4 mm vs 20.8 mm) and from tumor border (p = 0.007, median distances 15.4 mm vs 3.8 mm vs 0 mm) (Fig. 2b).

Tumors with IDH mutation and 1p19q co-deletion showed similar distances from both SVZ and SGZ as the tumors with IDH mutation and no co-deletion. When analyzed for IDH mutation status alone (IDH mutated vs IDH wild-type) we found no differences when it came to distance to the SVZ from neither the tumor center (p = 0.055, median distance 18.7 mm vs 14.9 mm) nor tumor border (p = 0.752, median distance was 0 mm for both groups) (Fig. 2c). There were differences between the IDH mutated and IDH wild-type gliomas with respect to the SGZ, both when measured from tumor center (p < 0.001, median distance 35.7 mm vs 20.8 mm) and tumor border (p = 0.006, median distance 7.7 mm vs 0 mm) (Fig. 2d).

Frontal lobe location was prevalent among all LGGs with 53% (84 of 159 tumors) of tumors and among these 82% (69 of 84 tumors) were IDH mutated. Temporal lobe harbored 22% of all LGGs (35 of 159 tumors) and among these 43% were IDH wild-type (Table 3).

**Table 3:** The distribution of types of LGGs across anatomical regions

	Oligodendroglioma (n = 49) IDH mutated, 1p19q co-deleted	Astrocytoma (n = 65) IDH mutated, 1p19 non-co-deleted	Astrocytoma (n = 45) IDH wild-type
Frontal	63% (31)	58% (38)	33% (15)
Parietal	14% (7)	11% (7)	11% (5)
Temporal	14% (7)	20% (13)	33% (15)
Deep central	8% (4)	11% (7)	20% (9)
Brainstem/cerebellum	0% (0)	0% (0)	2% (1)

Note that results are given as percentage of type-specific total (number of cases in parentheses)

## Discussion

In this population-based cohort of previously untreated low-grade gliomas, we found that LGGs are more often found closer to the SVZ than the SGZ. However, IDH wild-type tumors are more often found near the SGZ. These findings may suggest that the stem cell origin of IDH wild-type and IDH mutated LGGs may be different.

Previous studies on the distribution of molecular subtypes in gliomas have used a lobe-oriented approach that does not necessarily reflect the stem-cell theory of origin in gliomas [1]. Even so, it has been shown that LGGs in general are preferentially located near the insula [22] and are rarely found in the posterior regions of the brain [23, 24]. IDH-mutated gliomas are more often located in the frontal lobes, specifically around the rostral extension of the lateral ventricles [25–27], a location also seen in IDH-mutated glioblastomas [28, 29]. IDH wild-type tumors are more often located in diencephalon or brainstem [30]. We found that frontal location was more common with IDH-mutated tumors both with and without 1p19q codeletion, as previously shown [31, 32]. As the presence of 1p/19q co-deleted tumors is limited to a subset of tumors with IDH mutations they show the same anatomical predilection as IDH-mutated tumors in general.

Only one previous study by Tejada Neyra et al. have looked at molecular markers in gliomas and their relation to neurogenic niches [27]. The exploratory study of Tejada Neyra et al. included 131 LGGs in addition to 237 glioblastomas and found the rostral extension of the lateral ventricles to be a potential location for the cell of origin in IDH mutated gliomas [27]. Our study specifically looked at the positioning of tumors in relation to the neurogenic niches and confirmed the close relation to the ventricles in IDH mutated tumors. In addition, we identified a potential origin for IDH wild-type gliomas in the SGZ.

Recent evidence suggests that the SVZ may harbor the cell of origin in IDH wild-type glioblastomas [8] and thus proximity to this location might be expected for such gliomas. The SGZ and the SVZ differ in both anatomical location and histology; while the SVZ is close to the ventricles and the NSC of this niche give rise to both oligodendrocyte and neuronal lineages, the SGZ is tucked away in the hippocampus and their NSC give rise exclusively to the granular cells of dentate gyrus [33]. If the NSC is the cell of origin in all gliomas, this might explain our finding regarding the spatial relationship to the SGZ. However, the cell of origin is still debated, and another resident progenitor cell, the oligodendrocyte precursor cell (OPC), found in the SVZ and widely distributed in the cortex but not in SGZ, is also a possible cell of origin [34].

Several previous studies examining the anatomical location of LGGs were done before the 2016 revision of the WHO-classification system [26, 28, 30, 35–38] and results from these studies are not directly transferable to the current classification system of gliomas as some of them included oligoastrocytomas, a diagnosis that no longer exists [39]. Also, only a few studies in recent years have assessed both IDH1 and IDH2 mutation status in relation to anatomic location [27, 30, 32].

A strength in the present study is the population-based inclusion. The two university hospitals in this study have strict regional-based referral, which limits the potential for selection bias. Most previously published reports have evaluated tumor location from subjective assessment of 2D images. Quantitative analyses of the 3D distribution of tumors in MRI based maps as used in the present study, are less subject to classification or confirmation bias. The shortest distance from tumor to an anatomical structure is problematic to assess in 2D images, and this assessment usually results in an overestimation of the distance.

Gliomas tend to infiltrate white matter tracts and along vessels and the shortest distance as measured in the present study may not reflect the tumor migration pattern. As illustrated in a recent publication from our group, tumor progression in glioblastoma was often parallel and seldom perpendicular to major white matter tracts [40]. Gliomas are capable of fast migration and it is possible that other unknown factors may influence both speed and direction of tumor migration. As the true migrated distance from neurogenic niches to the radiologic border and core of the tumor is impossible to measure, shortest distance in 3D maps was assessed in the present study.

A possible confounder in our study is tumor volume, as a large tumor could show proximity to any region. However, IDH wild-type gliomas in our cohort were smaller than IDH mutated gliomas. Thus, tumor volume does not explain the short distance from the SGZ to IDH wild-type lesions. The frontal predilection of IDH mutated tumors may in part explain our findings as the distance to the SGZ is intrinsically longer for this group. But, as the SVZ has been suggested as potential location of origin for IDH wild-type tumors, this does not explain our findings regarding the SGZ.

A possible source of classification bias is the presence of other neuroglial tumors in our cohort. Tumors such as gangliogliomas or dysembryoplastic neuroepithelial tumors may be mistaken for diffuse WHO grade II LGG in small tissue samples. Both these WHO grade I tumors have a predilection for temporal lobe and do not carry an IDH mutation [39]. This potential misclassification is unlikely as the material has been thoroughly reviewed as previously described [41] and the incidence of these tumors is even lower than for low-grade gliomas.

A limitation of our study is the heterogeneity of the different sequences in MR protocols and variations in the interslice gap which may have influenced the accuracy of the segmentations. This is partly due to the retrospective nature of this study showing slightly differing tumor imaging protocols between the hospitals, but also the evolution of clinical brain tumor imaging going from 2D FLAIR to 3D FLAIR sequences.

Registration of a MR image with a tumor to a template based on healthy brains is associated with registration inaccuracies. This is more notable in tumors with a significant mass effect such as compression of the ventricles or midline shifts. Because of this, our Fig. 1 show some tumors to be located within the ventricles. Forcing the tumor outside the ventricle would impact the registration accuracy in other regions. We have therefore chosen to leave the tumors in the ventricles as this gives the most accurate impression of the actual tumor distribution.

The distribution of mutational status in our cohort is similar to what is published in other studies [42]. It should be acknowledged that diffuse WHO grade II LGGs is a heterogenous group and among the subgroup of IDH wild-type gliomas we find both “early glioblastomas” and entities with a more benign course [43]. If we had sufficient tissue, a subclassification of IDH wild-type lesions including TERT promoter methylation, EGFR-amplifications or combined whole chromosome 7 gain and whole chromosome 10 loss (+ 7/ − 10) could have been performed [44]. Also, whole-genome DNA methylation profiling of the whole cohort would have been of interest.

For this study, we used well-established molecular parameters in line with the current WHO classification of 2016 to determine complete IDH mutation status for almost all patients. For 1p19q codeletion status ATRX immunohistochemistry was used as a surrogate marker in a subset of patients. Here, IDH mutant tumors with ATRX loss on immunohistochemistry was assumed to be 1p19q intact. Loss of ATRX expression is close to being mutually exclusive to 1p/19q codeletion [45], but this approach might have caused a slight underclassification of 1p19q co-deleted tumors. In addition, a small subset of tumors additional sequencing for other IDH-mutations than IDH1 R132H was not feasible. None of these patients were 1p19q co-deleted and were assumed to be IDH wild-type. Although non- IDH1 R132H mutations are rare, this may have influenced our findings.

The neurogenic niche may harbor potential cells of origin for glioma formation, but in addition the neurogenic niche itself is a system promoting proliferation and migration, and may therefore play a role in tumor cell migration, tumor maintenance and even recurrence [7]. It has therefore been speculated that glioma cells may migrate *towards* the neurogenic niches to take advantage of an already established system. This may also imply that the origin of gliomas lies elsewhere. Although our study is not equipped to answer this question, radiographic studies do show interesting patterns in glioma distribution related to the neurogenic niches: Glioblastomas with close contact to the SVZ are more likely to be multifocal and more invasive [46] and contact with the SVZ is a negative prognostic factor [47]. In contrast, contact with the SGZ does not seem to influence survival [48]. For low-grade astrocytoma, it has been reported that anatomical involvement of the SVZ might predict poor survival [49, 50]. Still, due to cell migration it is possible that the origin of gliomas is not related to the center of maximum tumor load.

In conclusion, our study confirms the close anatomical relationship between low grade gliomas and the SVZ, as found in previous studies. We found that IDH status predicts distance to the SGZ as the IDH wild-type tumors are more often found near SGZ. This may imply that IDH wild-type gliomas are of a different anatomical origin than IDH mutated gliomas.
